# Need Analysis of Clinician-Oriented Integrated Precision Oncology Decision Support Tools: Qualitative Descriptive Study

**DOI:** 10.2196/67476

**Published:** 2025-09-08

**Authors:** Sheng Zheng, Yi Feng, Jieran Long, Xiang Li, Meng Zhang, Hui Chen, Xue Du, Huilong Duan, Shuqin Jia, Nan Wu, Xudong Lu

**Affiliations:** 1 College of Biomedical Engineering & Instrument Science Zhejiang University Hangzhou China; 2 Key laboratory of Carcinogenesis and Translational Research (Ministry of Education/Beijing) Center for Molecular Diagnosis Peking University Cancer Hospital & Institute Beijing China; 3 Department of Thoracic Oncology Peking University Cancer Hospital & Institute Beijing China; 4 Key Laboratory of Carcinogenesis and Translational Research (Ministry of Education/Beijing) Department of Thoracic Surgery II Peking University Cancer Hospital & Institute Beijing China; 5 State Key Laboratory of Holistic Integrative Management of Gastrointestinal Cancers Beijing Key Laboratory of Carcinogenesis and Translational Research Center for Molecular Diagnosis Peking University Cancer Hospital & Institute Beijing Beijing China; 6 State Key Laboratory of Molecular Oncology Beijing Key Laboratory of Carcinogenesis and Translational Research, Department of Thoracic Surgery II Peking University Cancer Hospital & Institute Beijing China

**Keywords:** precision oncology decision support, needs analysis, structured observation, semistructured in-depth interview, functional framework, artificial intelligence

## Abstract

**Background:**

The rapid advancement of next-generation sequencing has significantly expanded the landscape of precision medicine. However, health care professionals face increasing challenges in keeping pace with the growing body of oncological knowledge and integrating it effectively into clinical workflows. Precision oncology decision support (PODS) tools aim to assist clinicians in navigating this complexity, yet their current functionalities only partially address clinical needs. A lack of comprehensive needs assessment may result in unaddressed requirements, limiting the effectiveness of these tools in real-world practice.

**Objective:**

This study aimed to explore clinicians’ needs and expectations regarding the functionalities of integrated PODS tools, providing insights into essential features that could enhance their usability and impact.

**Methods:**

We conducted a qualitative investigation at Peking University Cancer Hospital to explore clinicians’ needs and expectations for the functions of integrated PODS tools. Data were collected through 143 structured participant observations during multidisciplinary team meetings and 17 in-depth semistructured interviews with a diverse group of oncology specialists, including physicians, surgeons, molecular biologists, radiotherapists, radiologists, and pathologists. Thematic analysis was applied to identify key functional requirements, and a requirements framework was formed.

**Results:**

Three overarching functional needs emerged: (1) better access to oncological knowledge, including support for therapy selection (guidelines, conferences, and consensuses), clinical trials, drug and treatment information, and complex case knowledge, as well as improved diagnostic and prognostic insights; (2) clinical contextualization and resource navigation, referring to the process of contextualizing scientific knowledge within real-world clinical settings, including access to clinical trials and drugs, along with predictive models for treatment response; and (3) support abilities in the decision-making process, highlighting the need for integration of flexible biological knowledge and phenotypic data; automated patient information synthesis; improved data visualization; and optimized retrieval, recommendation, and question-answering functionalities. A functional framework for integrated PODS tools was proposed based on these findings.

**Conclusions:**

The study conducted a qualitative descriptive observation and interview in the use, needs of integrated PODS tools. PODS tools serve as complex, multilevel decision support systems. A clear understanding of clinicians’ actual needs is crucial for their refinement and practical adoption. By capturing perspectives directly from oncology professionals, this study provides actionable insights into the functional enhancements required for PODS tools, ultimately aiming to bridge the gap between genomic advancements and clinical decision-making in precision oncology.

## Introduction

### Background

Advancements in next-generation sequencing have paralleled the rise of precision oncology, leading to the discovery of numerous diagnostic biomarkers for cancer and the approval of many targeted drugs by regulatory authorities, such as the US Food and Drug Administration, European Medicines Agency, and National Medical Products Administration, as well as recommendations by prestigious organizations such as the National Comprehensive Cancer Network), European Society for Medical Oncology, and Chinese Society of Clinical Oncology (eg, tepotinib [1] and sunvozertinib [2]). Despite this progress, health care professionals face challenges in keeping pace with these advancements and determining the most effective treatment strategies, as well as handling complex genomic information, such as regularly updating information and understanding the clinical significance of genomic alterations [3].

Currently, clinicians mainly rely on genetic testing reports to acquire the genomic profile of patients with cancer and to understand the clinical significance of certain genetic variants [4]. With rapidly growing knowledge of precision oncology, the content of genetic testing reports has greatly expanded, which also brings the problem of inconvenience in reading and searching [5]. Moreover, the heavy clinical workload significantly limits the time available to review the full contents of genetic testing reports [6]. A fast and effective way to display comprehensive genomic information may help clinicians locate the needed information easily [7].

Under these circumstances, computer-based interactive precision oncology decision support (PODS) tools have emerged. In oncology, tools with high interactivity and interoperability, such as knowledge and clinical trial (CT) databases [8-10], therapy recommendation engines [11], literature search tools [12], and predictive algorithm–based models [13], have been developed and are anticipated as valuable resources for facilitating the timely update and informed interpretation of genomic data from bioinformatics research to clinical statistical analysis [14-17]. The literature has described the properties, opportunities, and future effects of these tools [18,19].

However, it should be noted that although these tools may bring broad prospects in oncology research, they may not fully meet the needs of practical clinical application [20]. There are already systems integrating some of these functions of PODS tools for clinicians [21,22], but 2 key issues of current clinician-oriented PODS tools remain unclear.

The first issue is that clinician-oriented PODS functions are scattered across different tools. When somatic mutations and germline variants were detected in patients with cancer, to make a more appropriate and individualized treatment plan based on the patients’ genomic profile, some functions, such as (1) whether these variants are pathogenic or oncogenic [23], (2) how they are involved in tumorigenesis and cancer progression [24,25], (3) what their biological functions and clinical significance are [26,27], (4) to what extent they potentially affect the patients’ diagnosis and treatment [28], and (5) whether they will influence the choice of certain therapeutic strategy or clinical management, may be clinicians’ interests [29-32]. However, currently, a single available knowledge base could only meet 1 or 2 aspects of the abovementioned clinical requirements. To answer all these questions, clinicians must search in multiple knowledge bases using various search engines to achieve 1 goal, as different evidence may be scattered across diverse databases [33]. Such a process of switching tools normally costs a large amount of time with limited achievement.

The second issue is that some clinician needs regarding PODS tools remain unclear. Clinicians from different specialties may require different PODS functions, and while some commonalities could be found through needs analysis, the current lack of need analysis results in current PODS tools not yet meeting all practical requirements from clinicians. This fact restrains the clinical implementation of PODS tools and underestimates the clinical value of genomic data from patients with cancer [7]. Moreover, the lack of need analysis exposes the problem that there is currently no appropriate evaluation metric for the measurement of the quality of developing such metrics for PODS tools [34].

### Objectives

This study aimed to explore clinicians’ perceptions of functional needs in PODS tools. It also seeks to identify the existing urgent needs not met by current tools and outline a functional need framework for PODS tools.

## Methods

### Study Design and Theoretical Framework

This qualitative descriptive study, informed by a naturalistic perspective and grounded theory [35,36], investigates how clinicians use PODS tools in multidisciplinary teams (MDTs) and routine clinical practice. Comprising experts such as molecular biologists, physicians, surgeons, radiotherapists, radiologists, pathologists, and specialists from other departments (Table 1), MDT plays a crucial role in integrating clinical data and molecular profiling to tailor targeted therapies [37-39]. In routine clinical practice, such as in outpatient services, clinicians see many patients with simple to complex medical conditions. When the course of disease and treatments has either no clinical guideline to be followed or lacks evidence to support standard treatments, the case is raised for discussion in an MDT. Therefore, structured observation of MDT discussion was selected to investigate the need for PODS tools for clinicians and to propose elements for a framework of PODS tools. These elements were then validated and further verified by in-depth semistructured interviews, which might not only reflect the perspective of clinicians and quickly capture their views as a complement to objective observation but also constantly explore the deep reasons behind the ideas for unclear clinical requirements.

**Table 1:** Characteristics of MDT^a^ records.

Site			Thoracic Oncology Center		Gastrointestinal Oncology Center
Observations, n			26		20
Records, n			86		57
Participants, n			15-40		10-20
Patients with genetic testing, n (%)			39 (45)		12 (21)
Frequency			Once a week		Once a week
Duration (min)			10-120		20-40
Discussion duration per patient (min)			10-20		10-15
Speakers per discussion, n			4-10		3-6
**Participating specialties**					
	Surgeon	✓		✓	
	Physician	✓		✓	
	Radiotherapist	✓		✓	
	Radiologist	✓		✓	
	Pathologist	✓		✓	
	MDC^b^ staff^c^	✓		✓^d^	
	Nursing	✓		—^e^	
	TCM^f^	✓		—	

^a^MDT: multidisciplinary team.

^b^MDC: Molecular Diagnostic Center.

^c^Molecular biologists and geneticists from the Molecular Diagnostic Center.

^d^The molecular biologists and geneticists are present, but because of the genetic test information from the patients, they seldom speak in multidisciplinary teams and the Traditional Chinese Medicine Department.

^e^Not applicable.

^f^TCM: traditional Chinese medicine.

The study adhered to the COREQ (Consolidated Criteria for Reporting Qualitative Research) checklist [40]. Conducted from June 2023 to June 2024, the study used a multiple qualitative approach, combining structured participant observation and in-depth semistructured interviews to comprehensively assess the use and integration requirements of PODS tools [41].

### Setting and Data Collection

Structured participant observations and in-depth semistructured interviews were conducted at Peking University Cancer Hospital (PUCH). Participant observation data were collected during MDT meetings held by the Thoracic Oncology Center and Gastrointestinal Oncology Center; these meetings occurred weekly and involved large discussion groups, with an average of 19.92 (SD 5.51) participants per session.

**Figure 1:**
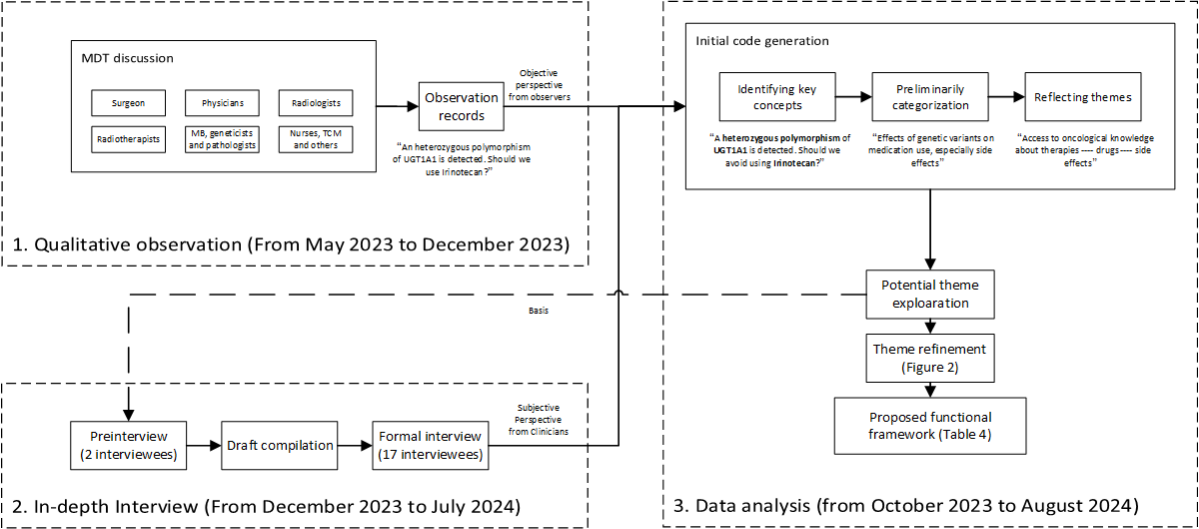
The process of the research. MB: molecular biologist; MDT: multidisciplinary team; TCM: traditional Chinese medicine.

The process of the research could be divided into 3 parts: qualitative observation, in-depth semistructured interview, and data analysis (Figure 1). MDT principals and interviewees all signed informed consent. Interviewees were selected using sampling theory and snowball sampling [42], based on the following criteria: (1) holding a medical certificate, (2) working in tumor-related clinical practice, (3) participating in MDTs, and (4) having experience using PODS tools or having heard of or observed the operation of PODS tools. Data saturation was defined as the point at which <5% of the total codes were new across 3 consecutive MDT observations. Snowball sampling was terminated when no new codes or ideas emerged from the 2 most recent interviews. Each interview took place at the clinicians’ lounge.

### Qualitative Structured Participant Observation

From May to December 2023, 2 team members, SZ and YF, observed MDTs at the Thoracic Oncology Center and Gastrointestinal Oncology Center. YF participated fully in MDT discussions as a member of the Molecular Diagnostic Center (MDC) and a participant-as-observer, while SZ, a doctoral candidate in medical informatics trained in qualitative research, took part as an observer-as-participant. The combination of the 2 observers balanced the perspectives of both clinical practice and informatics. SZ made preliminary records of the observations (Multimedia Appendix 1 [43-45]), and these records were subsequently refined in collaboration with YF. The MDT at PUCH can be divided into three parts: (1) patient information preparation before the MDT (*preparation*), (2) patient information briefing during the MDT (*briefing*), and (3) multidisciplinary discussion (*discussion*). The observation of this research mainly focused on the discussion part, which was recorded in detail to identify PODS needs related to genomic interpretation, diagnosis, therapeutic recommendation, and possible functional requirements of PODS tools that emerged during the discussion (a description of the phenomena, the process of MDTs, and detailed observation of the other 2 parts are provided in Multimedia Appendix 1). To ensure methodological rigor, we implemented a data saturation verification protocol. For proactive monitoring, we tracked emerging codes through real-time coding logs during observations and conducted weekly codebook audits using NVivo’s (Lumivero) code frequency matrices to ensure that it achieved semantic saturation [46]. The slides presented during MDTs were obtained after the meeting to refine the records. The team observed 26 Thoracic Oncology Center MDTs (86 records) and 20 Gastrointestinal Oncology Center MDTs (57 records), with each session lasting approximately 35 minutes on average.

### In-Depth Semistructured Interview

Semistructured, in-depth qualitative interviews were conducted from December 2023 to July 2024 by SZ and YF, with SZ serving as the main interviewer and having no prior interaction with the participants. YF, a colleague of the interviewees, assisted SZ in refining and complementing questions. The semistructured interview questions were developed based on the potential themes that emerged from the initially coded observation record data. Before the formal interview, 2 interviewees were invited to the preinterview session, during which the draft interview guide was compiled based on the initial coding of the qualitative observation records. Both interviewers maintained reflexive journals to document emergent assumptions and field observations. The formal interview guide was later standardized to focus on clinicians’ use of genetic test reports and their needs and expectations for PODS tools (Multimedia Appendix 1). Clinicians were encouraged to provide additional perspectives on PODS and to share their experiences using PODS tools through open-ended questions during the interview. After each interview, potential themes were extracted. If data saturation had not been reached, additional interviewees were recruited. The demographic details are summarized subsequently. The interviews were conducted in Chinese, audiotaped with a digital recorder, transcribed verbatim, and edited by SZ. A total of 12 hours of individual interviews were transcribed, resulting in approximately 48,000 words. The interviews lasted approximately 40 (range 26-70) minutes.

### Data Analysis

The observation and interview data were thematically analyzed following the reflexive thematic analysis by Braun and Clark [47], which includes familiarization with the data, generating initial codes, exploring potential themes, reviewing themes, defining and refining themes, and finalizing findings. The data analysis was divided into 2 steps. Between the observation and interview phases, the observation record data were initially coded, and the potential themes that emerged formed the basis of the interview. After each interview, the themes explored in the interview were compared with the initial potential themes as a form of constant comparison and validation [38]. This was considered part of the review process. Subsequently, the 2 classes of themes were combined and refined. The functional framework of PODS tools was extracted through the themes and concluded through a team discussion. The interviewees were invited to confirm whether their perspectives were fully represented during the construction of the framework. NVivo 14 software was used for the analysis, with coding performed by SZ and reviewed by YF. Cohen κ was calculated to confirm strong coding consistency. Discrepancies were resolved through team discussions.

### Ethical Considerations

The study received ethics approval from the ethics committee of PUCH (2023KT35). Informed consent was obtained from all interview participants for both the interviews and the secondary analysis of the interview data. All observation and interview data were recorded anonymously. The interviewees received a small thank-you gift of approximately CNY ¥50 (US $7.00) after the interview.

## Results

### Qualitative Observation and Interviewee Characteristics

**Figure 2:**
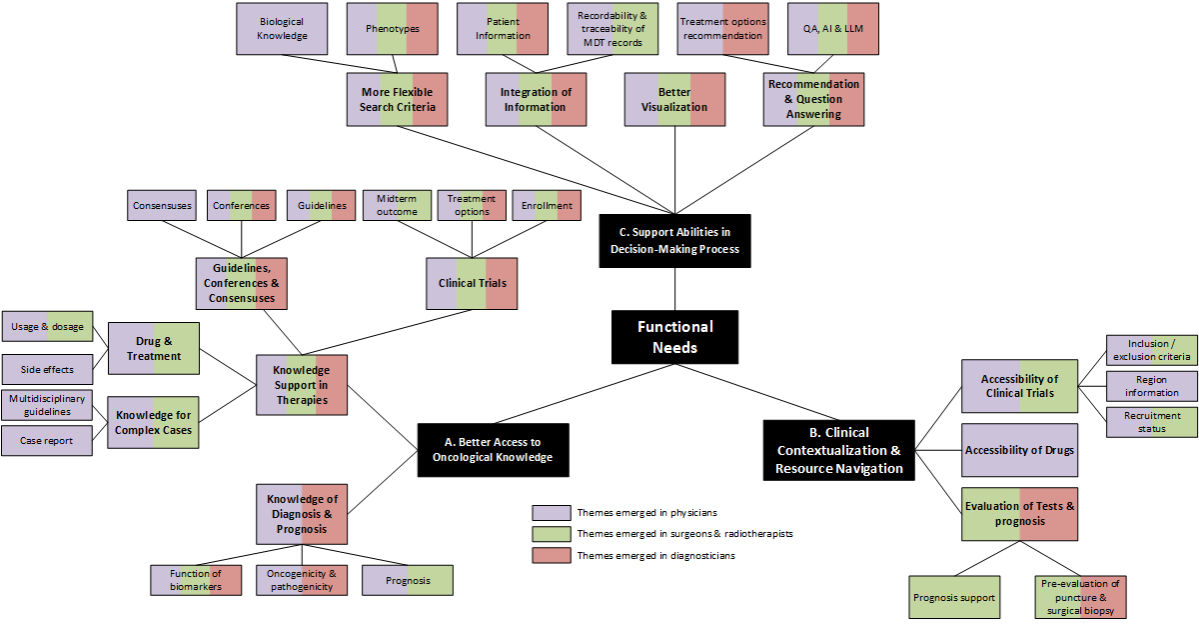
The 3 key themes emerging from the qualitative observation and interviews: (1) better access to oncological knowledge: clinicians emphasized the importance of precision oncology decision support (PODS) tools in facilitating access to high-level precision medicine evidence, including guidelines, treatment information (eg, use, dosage, and side effects), clinical trials, multidisciplinary guidelines, and case reports; (2) clinical contextualization and resource navigation: PODS tools aim to integrate fragmented clinical information, such as trial eligibility, drug accessibility, and evaluation models, enabling clinical contextualization and resource navigation for practical application; (3) support abilities in the decision-making process: clinicians anticipated that the PODS design would enhance decision-making efficiency. They also highlighted advancements in information systems, including user-friendly interfaces, enhanced search functions, chatbots, and large language model applications, as key features to improve workflow and clinical decision-making. AI: artificial intelligence; LLM: large language model; QA: question answering.

A total of 46 MDT discussions were qualitatively observed, involving treatment decisions for 143 patients (Table 1). For the 46 MDT observations, saturation was reached by the 41st MDT, and no new codes emerged in the 45th and 46th MDT (Figure S1 in Multimedia Appendix 1). After 6 months of observation, the research team began the first round of exploring potential themes. Potential needs for PODS tools emerged from our observations (Tables S2-S6 in Multimedia Appendix 1), which were refined and validated by the in-depth semistructured interviews and further constructed into the themes of functional needs in PODS (Figure 2). The MDT in the Thoracic Oncology Center had significantly more detailed information and longer discussion times for genetic testing and personalized targeted therapy compared to the MDT in the Gastrointestinal Oncology Center. In addition, the use of PODS tools was more prevalent in the Thoracic Oncology Center.

On the basis of these findings, we focused our interviews with clinicians in the Thoracic Oncology Center on their use of PODS tools. We also conducted interviews with clinicians from the Radiotherapy Department and the MDC.

A total of 17 clinicians participated in in-depth semistructured interviews (Table 2). For the 17 interviews, saturation was achieved by interview 15, with no new codes appearing in interviews 16 and 17 (Figure S2 in Multimedia Appendix 1). All 17 clinicians had experience using genetic testing reports, while 59% (n=10) had personal experience using at least 1 interactive PODS tool. However, all 17 clinicians had heard of or observed the use of PODS tools. The potential needs were screened and confirmed during the interview. After data analysis, 3 themes emerged from our observations and interviews (Figure 2; Figures S3 and S4 in Multimedia Appendix 1; and Tables S2-S6 in Multimedia Appendix 1). Themes that emerged from the interview data and were organized by knowledge, feasibility, and support ability levels are described subsequently and presented in Table 3. From these 3 themes, the functional framework of integrated PODS tools is described in detail in the subsequent sections.

**Table 2:** Characteristics of the interviewees (N=17)^a^.

Characteristics			Interviewees, n (%)
**Sex**			
	Male	10 (59)	
	Female	7 (41)	
**Years of working**			
	<10	6 (35)	
	10-20	8 (47)	
	>20	3 (18)	
**Clinical duty**			
	Physician	9 (53)	
	Surgeon	5 (29)	
	Molecular biologist	2 (12)	
	Radiotherapist	1 (6)	
**Professional title**			
	Chief	2 (12)	
	Associate chief (deputy chief)	7 (41)	
	Attending chief (supervising)	5 (29)	
	Attending	3 (18)	
Preparer			6 (41)
Had used at least 1 PODS^b^ tool			10 (59)

^a^The characteristic “preparer” refers to a role in the multidisciplinary team, responsible for collecting patient information, preparing multidisciplinary team briefing slides, and presenting the information during the meeting. Further description of the preparer is provided in Multimedia Appendix 1.

^b^PODS: precision oncology decision support.

**Table 3:** Sources of themes from key informants, translated into English.

Theme and subtheme				Illustrative quote and quote number
**Better access to oncological knowledge**				
	**Knowledge support in therapies**			
		Guidelines, conferences, and consensuses	“Before the MDT, Dr. [name] would like to share recent progress of [progress in treatment of specific disease]...” [Observation 008, 028, and 079] (Quote 1) “The way I get access to the oncological knowledge are usually from WeChat official accounts, going to conferences, and newly updated information shared by peer expertise. Sometimes I also search for literatures or reviews in my spare time...What I’m most interested in is the change of the latest version update guideline, from which I could learn a lot...would be great if there is a notification of the changes...” [005, associate chief physician] (Quote 2) “We usually discuss the latest progress of conferences before the MDT starts. If PODS tools could notify us this information in our spare time, it would help a lot in summarizing this information for discussion...” [006, associate chief physician] (Quote 3)	
		Clinical trials	“About 25-50% of patients in our hospital would require enrollment in CTs...develop a real-time updating CT recommendation tool is the best...” [004, attending physician] (Quote 4) “...The information in the website of CTs updates frequently...requires us to retrieve and summarize the latest eligibility criteria and enrollment every half month to one month...so time-consuming...” [009, associate chief surgeon] (Quote 5) “...Latest progresses are often discussed in MDT...not so convenient to check...we want future PODS tools could capture these in real time...” [005, associate chief physician] (Quote 6) “Before the MDT, Dr. [name] would like to share the midterm result of [progress of specific CT] for us...” [Observation 033 and 082] (Quote 7)	
		Drugs and treatment options	“Genetic test reports that vormetinib is effective in LUAD patients with EGFR exon 20 insertion mutation...the descriptions are not particularly clear...we sometimes want to understand the mechanism of drugs...” [003, chief surgeon] (Quote 8) “Osimertinib, vormetinib and almonertinib are commonly used among the third generation of EGFR-TKIs...there are differences in their use, which requires years of clinical experience...We hope PODS tool will support us in this regard...” [010, attending physician] (Quote 9) “...Some drugs are needed for four cycles, but some patients’ health condition is poor...We want to know whether can I reduce the dosage...sometimes drug suppliers would tell us some information, but that’s not enough...” [005, associate chief physician] (Quote 10) “...The two most common and risky side effects are vomiting and leukopenia...need prompt intervention...if PODS tools could have an alert or reminder function of these side effects, it would help us reduce unnecessary prescriptions to alleviate these side effects...” [001, associate chief physician] (Quote 11)	
		Knowledge for complex cases	“...when the patient has other cancers...we would invite colleagues from the corresponding departments for MDT discussion, but it is brief...If we can learn easily from other disciplines, we can make treatment decisions more quickly...” [006, associate chief physician] (Quote 12) “Today’s discussion is about the patient with malignant pleural mesothelioma for whom conventional chemotherapy drugs did not respond...if any doctors have seen relevant case reports?” [Observation 046] (Quote 13)	
	Knowledge support in diagnosis and prognosis		“...some colleagues asked me the differences between germline variants and somatic mutations...PODS tools could be a useful way to help my colleagues understand the biological knowledge like this...” [014, supervising molecular biologist] (Quote 14) “...we sometimes want to know the biological function of molecular alterations, understanding why the alteration happens could better guide us to understand the mechanism of the disease...” [013, associate chief surgeon] (Quote 15) “...previously encountered a LUAD patient with co-mutations in TP53, RB1, and EGFR...no appropriate standard treatment or diagnosis options but continued observation at the time...an article said that the concurrence of three variants may be induced to small cell cancer transformation, which is an important reference basis to do puncture for diagnosis...hope to have a better mechanism to search evidences...” [011, attending physician] (Quote 16^a^) “...we often evaluate the prognosis between different therapies, like radiotherapies and targeted therapies...the knowledge of which could help us make decision better...” [017, attending chief radiotherapist] (Quote 17)	
**Clinical contextualization and resource navigation**				
	Accessibility of CTs^b^		“When there is a patient needs to be enrolled, we will look at the inclusion and exclusion criteria in the CTs we have printed (take out some hard copy of CTs) and compare the patient’s medical status with the criteria item by item...it is very time consuming...Our CT screening usually follow this sequence: ‘First, CTs held in our ward; Second, CTs held in our hospital; Third, CTs held in Beijing’s Hospital; Fourth, different places other than Beijing in China; Lastly, we consider overseas’...CT enrollment is usually based on the screening order of inside the department, hospital, Beijing, China and foreign countries...We need to manually screen it ourselves...want an automatically sort...” [002, attending surgeon] (Quote 18) “Sometimes we know there is an enrollment of CT, but we don’t know the up-to-date recruitment status. Future PODS tools or CT website should update the status more frequently.” [006, associate chief physician] (Quote 19)	
	Accessibility of drugs		“...Osimertinib is covered in Drug Catalog, but vormetinib and almonertinib need to be paid at patients’ own expense...” [001, associate chief physician] (Quote 20) “Clear mark of drug catalog and price information of drugs can make us discuss the most suitable treatment option for with patient him/herself.” [012, associate chief physician] (Quote 21)	
	Prediction models: the necessity of ordering diagnostic tests and prediction of prognosis		“...some genetic tests are expensive...I have known that there are some radiology technologies to auxiliary identify whether there is a certain risk of mutations...” [012, associate chief physician] (Quote 22) “...examinations like puncture examinations are invasive...we want to use PODS tools to help estimate the necessity of such invasive examinations...” [013, associate chief surgeon] (Quote 23) “...common mutations like TP53 have a poor prognosis...We want to combine the molecular alteration with prognostic prediction...” [002, attending surgeon] (Quote 24)	
**Support abilities in the decision-making process**				
	Using more flexible biological knowledge and phenotypes as search criteria		“...we once had a patient with MEK mutation and ERK wild type...there were not many drugs targeting MEK...wanted to find some CTs targeting ERK...had to reset the search criteria which was inconvenient...would like to have a flexible retrieval mechanism...” [008, associate chief physician] (Quote 25) “...would be great if PODS tools could automatically match the inclusion criteria with patient’s information and tell us directly whether this patient is a qualified candidate...helps us reduce a lot of comparison work...” [007, chief physician] (Quote 26)	
	Automatic integration of patient information		“...would be great if we just input the ID of patient and click a button...all the information scattered in HIS, PACS...is organized...” [011, attending physician] (Quote 27) “Acquiring the radiology information is inconvenient currently. We have patients all over the country, and they have a variety of formats and types of image information. We hope that the future of PODS tools could help us to reduce the burden of identification and input of these data.” [010, attending physician] (Quote 28) “Suppose there is a timeline of patient in MDT, and all the events of this patient are on the line. Click one button and the details of the event will be shown. That’s amazing...” [002, attending physician] (Quote 29) “Some patients will be discussed for several times. We want the PODS tools to record our discussion every time.” [004, attending physician] (Quote 30) “This patient was discussed [time] ago. Last time we decided to use [treatment option], but the disease progressed now...” [Observation 033] (Quote 31)	
	Better visualization of information		“...Switching slides back and forth to watch the texts is inconvenient...hard to sort out the therapeutic relationships...” [010, attending physician] (Quote 32) “...automatically generated timeline of history is more logical and visualizable...” [002, attending surgeon] (Quote 33) “...when there is a patient with rare mutation...may want to find previous patients who have the same or similar mutation...may be helpful for diagnosis and treatment...some simple automated generated statistical distribution plots are intuitive to see and discuss...” [014, supervising molecular biologist] (Quote 34)	
	Optimization in retrieval, recommendation, and question answering		“...would be great if PODS tools could automatically match the inclusion criteria with the patient information...” [005, associate chief physician] (Quote 35) “...don’t have enough time to read and search the evidences...we may just input some key search information to AI, and ask what latest information it finds...” [015, associate chief physician] (Quote 36) “I wonder if there’s a day if AI could be a real assistant for me. Nowadays there is chatGPT, I believe in future there will be a chatbot in oncology area, knows everything, tells us what is the best treatment option...future PODS tools may be like this...” [002, attending surgeon] (Quote 37)	

^a^The interviewee referenced the study by Lai et al [43].

^b^CT: clinical trial.

The percentages in brackets in the sections below represent the percentage of interviewees who reported the corresponding functional requirements. The percentages reflect only the proportion of participants who mentioned a specific need during the interviews and do not indicate the relative importance of the needs.

### Theme 1: Better Access to Oncological Knowledge

Oncological knowledge plays an important role in PODS, and the most frequently mentioned need for PODS was faster and better access to the latest oncological information. Precisely, comprehensively, and timely integrating the knowledge of diagnosis, treatment, and prognosis from various sources, such as knowledge bases, academic conferences, and guidelines, is the most valuable function of PODS tools.

#### Knowledge Support in Therapies (17/17, 100%)

All interviewees emphasized the importance of the function of knowledge acquisition for therapies in support of clinical practice in oncology.

##### Guidelines, Conferences, and Consensuses (12/17, 71%)

Clinicians paid much attention to the latest progress in oncology and updates released in guidelines, conferences, and consensuses. However, the current access to this information was mainly through manual search and information shared by colleagues (quote 1). To meet these requirements, a news update session is held before the MDT discussions in the Thoracic Oncology Center to review and interpret the latest updates released at academic conferences or by professional associations. However, once-a-week knowledge updates provided by the attending MDT may not meet the urgent needs during daily clinical practice. Clinicians hoped that this latest information could be updated in PODS tools in a timely manner (quotes 2 and 3).

##### CT Need (14/17, 82%)

All physicians referred to this need. Physicians had a greater need in this aspect, as most patients in the Thoracic Oncology Department present with advanced-stage conditions and require systemic treatment. After the failure of guideline-recommended therapies, appropriate CTs had to be considered. Approximately 25% to 50% of patients would require enrollment in CTs (quote 4), necessitating that physicians have a comprehensive understanding of the available options. The 2 major channels to access up-to-date CT information were manual searches of CT registration websites and information shared by physicians responsible for CT recruitment and data collection. However, frequent retrieval of this information imposed a burden on their workload. Timely and automatic updates of CT information could help alleviate this burden (quotes 4 and 5). Midterm outcomes of CTs were also given more attention (quotes 6 and 7).

##### Drugs and Treatments (8/17, 47%)

Surgeons and radiotherapists hoped to access the latest knowledge on targeted therapy, immunotherapy, and chemotherapy drugs through PODS tools (quote 8). Physicians had greater demands for drug information, including their use and dosage (quotes 9 and 10). Moreover, clinicians paid close attention to the side effects of the treatment regimens. It was important to be aware of and informed about the potential side effects of certain treatment options for every patient. At present, the prediction of side effects for certain therapeutic strategies still relies on clinicians’ experience, while current PODS tools provide limited support (quote 11).

##### Knowledge for Complex Cases (4/17, 24%)

In some complex cases, physicians expressed a need to acquire more evidence, such as multidisciplinary guidelines and case reports. When encountering patients with a history of multiple primary cancers, convenient access to multidisciplinary guidelines was identified as an important need (quote 12). For example, a patient undergoing targeted therapy for lung cancer was found to have a mass during colonoscopy, but it remained unclear whether the lesion was a primary colorectal tumor or a metastasis from the lung cancer. Furthermore, clinicians hoped to find similar case reports when managing patients with rare tumor types (quote 13).

#### Knowledge Support in Diagnosis and Prognosis (6/17, 35%)

In addition, interviewees sometimes mentioned diagnostic and prognostic evidence as important for the selection of treatment options. Especially in the area of diagnosis, experts from MDC and the Pathology Department were often asked to explain the functions, oncogenicity, and pathogenicity of specific biomarkers and molecular alterations to interpret their potential clinical significance (quote 14). PODS tools could help collect and filter related evidence from various knowledge bases and assist in a more comprehensive explanation and interpretation (quote 15). Moreover, current PODS tools lack reliable search functions for these purposes (quotes 16 and 17).

### Theme 2: Clinical Contextualization and Resource Navigation

When a treatment option was acknowledged or proposed, evaluating its real-world applicability, including insurance coverage criteria, availability of CTs and drugs, and relevant prognostic models, was necessary. Much of this work was repetitive and could be automated by the PODS tools, thereby accelerating decision-making and improving care coordination.

#### The Accessibility of CTs (9/17, 53%)

Whether patients had the channel to access appropriate CTs was as important as meeting the inclusion and exclusion criteria (quote 18). Geographic barrier seemed to be an important influencing factor that might directly affect CTs’ accessibility (quote 18). Furthermore, the up-to-date recruitment status was another essential factor that could help clinicians filter the available CTs and improve the effectiveness of patients’ enrollment (quote 19).

#### The Accessibility of Drugs (5/17, 29%)

The patient’s affordability was 1 of the biggest factors, after medical condition, when considering treatment options (mentioned by all interview participants). The drugs mentioned in knowledge bases or clinical guidelines might not be practically accessible because of cost. In such cases, clinicians wanted to know whether the cost of certain therapeutic drugs could be covered by the Chinese medical insurance policy or by commercial insurance, and they considered patient affordability when making decisions about a reasonable therapeutic regime (quotes 20 and 21).

#### Prediction Models: Necessity of Ordering Diagnostic Tests and Prediction of Prognosis (4/17, 24%)

Currently, genomic information can be acquired through genetic testing of a peripheral blood sample. However, tissue samples are sometimes needed to obtain more precise test results. The methods for obtaining tissue are usually invasive, such as puncture, bronchoscopy, or surgery, which often cause pain to patients. Clinicians were cautious about ordering invasive and unaffordable tests and preferred a less expensive way to assess the need for these tests (quotes 22 and 23). In addition, some surgeons reported using prognostic prediction models to help estimate the risk of surgery (quote 24).

### Theme 3: Support Abilities in the Decision-Making Process

During MDTs, there was also a clear need for support in the decision-making process.

#### Using More Flexible Biological Knowledge and Phenotypes as Search Criteria (10/17, 59%)

Clinicians expressed a demand to incorporate biological knowledge, such as the function of variants or signaling pathways, into their searches for information (quote 25). They also wanted to combine phenotypes in the process of identifying better treatment options. The most frequently mentioned phenotypes were stage, number of treatment lines (ie, the treatment course), location of lesion, presence of multiple primary cancers, and physical conditions such as Eastern Cooperative Oncology Group score and complications. Automatic matching of patients based on phenotype and inclusion and exclusion criteria was also a frequently mentioned need (quote 26).

#### Automatic Integration of Information (8/17, 47%)

Summarizing information during the preparation phase was described as repetitive and time-consuming. All preparers mentioned that it would be beneficial to have an automatic patient information integration function (quotes 27-29). The ability to record the discussions in MDT was also considered a valuable function, as some patients were discussed several times. Tracking not only the patient’s diagnostic and treatment history but also the previous MDT discussion conclusion was seen as extremely helpful (quotes 30 and 31).

#### Better Visualization of Information (3/17, 18%)

Some clinicians noted the inconvenience of slide-based presentations, especially when a patient’s medical history was long and had to be spread across several pages. In such cases, it was not uncommon for gaps in the patient’s diagnostic and treatment information to occur. They preferred a better visualization of patient information (quotes 32 and 33). Some clinicians were willing to see an automatic chart display function added to PODS tools to show statistical results from previous real-world genomic data more clearly. This improved data display function could potentially assist clinicians in viewing cancer mutation profile and understanding their relationship with clinical phenotype, therapeutic effectiveness, and prognosis more directly. Furthermore, such a function could not only help clinicians optimize clinical decisions based on previous in-house clinical evidence but also support scientists in designing clinical studies based on objective analysis (quote 34).

#### Optimization in Retrieval, Recommendation, and Question Answering (5/17, 29%)

In addition to search optimization (mentioned in Better Access to Oncological Knowledge section), clinicians wanted PODS tools to rank treatment options and CTs intelligently and to adjust recommendations as search conditions change (quote 35).

The popularity of language models has led clinicians to hope that artificial intelligence (AI) could help interpret clinical evidence in the literature through question answering (quotes 36 and 37).

### The Categorization of the 3 Main Thematic Needs

**Figure 3:**
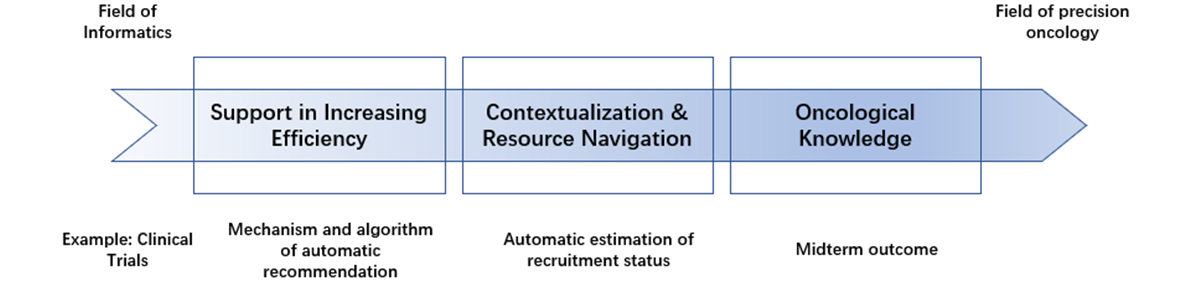
Precision oncology decision support–related needs following the “direction of flow” from the informatics domain to the precision oncology domain, highlighting the interdependence among the themes.

During the thematic analysis, we maintained a central objective: to leverage rapidly advancing information systems technology to support decision-making in precision oncology. This objective naturally extends to 2 fundamental scenarios: 1 focused on assisting clinicians in understanding precision oncology knowledge, which is more aligned with the medical field, and the other focused on directly using advancements in IT to help clinicians improve diagnostic and treatment efficiency, which leans more toward informatics. During the data analysis process, we found that clinicians may seek data unrelated to clinical knowledge but still critically important or infer implicit clinical decisions from the data. These 2 scenarios may lie at the intersection of medicine and informatics. On the basis of this, we categorized the 3 main thematic needs according to the “direction of flow” from the field of informatics to the field of precision oncology (Figure 3). These themes are hierarchically layered and functionally independent. The theme, *better access to oncology knowledge,* forms the foundational theme, supplying the raw, up-to-date evidence base. Another theme, *clinical contextualization and resource navigation*, builds on that knowledge by translating it into locally relevant, feasible options—evaluating insurance coverage, resource availability, and the patient’s socioeconomic context. The third theme, *decision support abilities in increasing efficiency*, leverages both themes to deliver actionable recommendations, visualizations, and workflow-integrated tools that streamline the clinician decision-making process.

### Variety of Needs Among Specialties, Professional Titles, and Roles in MDT

We found that clinicians from different specialties exhibit varying preferences in their needs (Table S3 in Multimedia Appendix 1), which are likely influenced by the nature of their departments and the types of patients they encounter. Physicians are more likely to treat patients with advanced or progressive-stage conditions, and therefore, their needs for oncological knowledge are most pronounced, particularly in areas such as CTs and the management of complex cases. In contrast, surgeons tend to prioritize PODS-related knowledge related to the perioperative period, such as surgical risks and postoperative complication assessments.

There are also varying needs based on different professional titles. For senior physicians, such as department heads, their extensive clinical experience allows them to make rapid diagnostic and treatment decisions. Thus, they expect PODS to provide more up-to-date knowledge, particularly regarding cross-disciplinary insights and the latest drug research. In contrast, younger physicians tend to seek more support in oncology pharmacology and case reports.

As for the roles in MDT, preparers (Multimedia Appendix 1) seek PODS to support the data preparation and interface presentation phases of MDT. They have a more extensive need for visualized data and convenient retrieval functionalities.

### The Functional Framework of Needs for Integrated PODS Tools

After the themes were extracted and confirmed, a functional framework of needs for integrated PODS tools was proposed. In the framework, the needs for PODS tools were basically divided into three dimensions: (1) to provide clinicians with up-to-date and straightforward oncology knowledge; (2) to enable clinical contextualization and resource navigation, offering suggestions for applicable and feasible clinical management; and (3) to offer comprehensive and efficient support for the decision-making process (Table 4).

**Table 4:** The functional framework of needs for integrated PODS^a^ tools.

Dimension and subcomponents		Functions of needs	Recommendation
**Up-to-date and straightforward oncology knowledge**			
	High-level evidence	Access to high-level clinical evidence (eg, guidelines, conferences, and consensuses) Concise overview of evidence, containing a general idea of medical information (eg, drug, therapy, and test results) Labeling of credibility or describing the clinical significance or referability Source of literature	High-level evidence (eg, guidelines, conferences, and consensuses) is always a priority for clinicians when making decisions about any patient. Clear labeling of the evidence level is also helpful for clinicians in determining the credibility of the evidence [44].
	CTs^b^	Information on inclusion and exclusion criteria (related to the accessibility of the CT function) Provide midterm results, if available, for CTs that are ongoing and not recruiting Provide enrollment information for recruiting CTs Comprehensive description of medication information as outlined in the CT protocol	After the failure of guideline-recommended therapies, appropriate CTs should be considered.
	Biological knowledge in diagnosis, treatment, and prognosis	Description of basic biological information of biomarkers, such as nomenclature (eg, alias and transcript numbering), type (eg, point mutation, fusion, and copy number variation), structural information (eg, mutation site and location in chromosome), and basic biological function Description of the medical function of biomarkers related to oncology, such as the role in tumor development (eg, oncogenic or tumor suppressor and driver or passenger), mechanism of involvement in tumor development (eg, kinase activation and DNA damage repair), and frequency of expression and mutation in different cancers Description of the medical function of biomarkers directly related to patient information, such as clinical significance (eg, diagnosis, treatment, and prognosis) and pathogenicity Description of inclusion in public evidence repositories of biomarkers Source of evidence	A better explanation of biological knowledge will help clinicians understand the pathological mechanism. The integration will also improve the interpretation of the clinical significance of genomic testing results [45].
	Drug and therapy information	Information on the use, dosage, and side effects of drugs and therapy options from prior medication and literature	The use, dosage, and side effects information of drugs and therapy options help clinicians implement treatment accurately.
	Case reports and preclinical evidence	Source of the case reports related to the patient Phase 1 to 2 CTs and animal experiments of drugs	Case reports and preclinical evidence are important references for complex or rare cases.
	Multidisciplinary guidelines	Clinical guidelines for different cancer areas	Although the proportion of patients with multiple primary cancers is small, the number of discussions about these patients is gradually increasing. These guidelines can be used to keep physicians updated on other cancer types outside of MDTs.
**Clinical contextualization and resource feasibility support**			
	Accessibility of CTs	Automatically match patient information with inclusion and exclusion criteria (refer to the information on the inclusion and exclusion criteria function) Automatically estimate recruitment status Provide an automatic geographic accessibility for eligible CTs	For patients, the enrollment criteria of CT are as important as the therapy itself. Letting the clinician know whether a certain CT could be accessed also matters.
	Accessibility of drugs and treatment options	Information on the insurance coverage status of drugs Cost of drugs and treatment options Approval information of drugs and treatment options	This function helps clinicians know about drug accessibility and could save time in screening available CTs and communicating with patients.
	Application of prediction models	Convenient interface to access prediction models	There are many prediction models in the oncology domain, such as operative risk assessment and risk of recurrence. However, the current PODS tools have not integrated these models yet. Integrating and applying these models could be helpful in decision-making.
**Comprehensive and efficient support abilities in the decision-making process**			
	Individualized search criteria	Incorporate biological knowledge (eg, the function of variants or signaling pathways) Incorporate phenotypic features (eg, stage, number of treatment lines [ie, the treatment course], and the location of the lesion) into search criteria	Optimization in search criteria, such as biological knowledge and phenotypes, could help clinicians search for treatment options more precisely.
	Automatic integration of patient information	Automatically summarize the patient’s diagnosis and treatment history Automatically integrate the patient’s health information from different systems in the hospital, such as the HIS^c^, PACS^d^, and LIS^e^.	The automatic summarization of patient information could help reduce the *preparation* time.
	Recordability and traceability	An interface for recording MDTf discussion, preferably automatically	A timely recording of the MDT discussion function could help clinicians trace back, summarize, and analyze previous data easily.
	Better visualization	Chronological patient history Logical display Organization of information by priority Clear hierarchy of information	Automatically generated timelines help clinicians read patients with a long history and complex treatment course straightforwardly, whereas graphs and tables can help them understand data better and interpret clinical significance more easily.
	Treatment option recommendation	Personalized ranking of drugs and treatments based on their advantages and disadvantages Provide automated treatment recommendations	A treatment recommendation function with evidence classification, data matching, and a sorting algorithm could provide extra comprehensive support to clinicians during decision-making.
	Question answering and dialogue	Automatic dialogue and question answering Large language model agents that automatically retrieve information or tools to support decision-making	The development of artificial intelligence and large language models may implement the function of multiturn question answering and dialogue, as well as provide precise information.

^a^PODS: precision oncology decision support.

^b^CT: clinical trial.

^c^HIS: hospital information system.

^d^PACS: picture archiving and communication system.

^e^LIS: laboratory information system.

^f^MDT: multidisciplinary team.

## Discussion

This qualitative descriptive study aimed to gain a more in-depth understanding of clinician-oriented integrated PODS tools. The data were collected with structured observations and semistructured in-depth interviews.

### Principal Findings

#### Needs in PODS

Three main themes of needs in PODS were extracted: (1) the *oncology knowledge* theme underscores the necessity of a continuously updated, structured knowledge resource that synthesizes genomic, therapeutic, and guideline data into clinically digestible formats; (2) the *clinical contextualization and resource navigation* theme highlights the importance of situating this knowledge within real-world practice to ensure that treatment recommendations are feasible and equitable; and (3) the *support abilities in the decision-making process* theme streamlines clinician decision-making and improve care efficiency.

#### Prospects for PODS Tools Based on the Proposed Framework

A functional framework for the needs of integrated PODS tools was proposed. Providing timely and updated oncology information was the most frequently mentioned need by clinicians. Currently, most clinicians still obtain the latest cancer knowledge through literature reviews; data released and presented at academic activities and conferences; or information shared by, for example, fellow experts and drug suppliers. An integrative PODS tool that properly meets the clinical requirements for up-to-date knowledge summarization and presentation is not yet available. However, clinicians are not satisfied with the tools that are currently available. The main issues include delayed updates, poor retrieval conditions, and insufficient integration with molecular biological evidence. PODS tools are supposed to incorporate the interpretation of available molecular testing results to meet the requirements of the precision medicine concept and to aid the molecular tumor board [21,22].

The framework suggests that integrated PODS tools related to oncology knowledge should include a timely updated and comprehensive knowledge base [48], incorporating drug approval information from official administrative authorities; clinical guidelines; and consensus from well-recognized organizations, released CTs’ results from academic conferences, preclinical evidences from literature reviews, and open CTs’ updates through official channels. This knowledge base should be highly structured and user-friendly to meet the advanced retrieval needs of clinicians, focusing on information regarding genetic variant data, molecular functional significance, evidence on therapies and prognosis, multidisciplinary guidelines, and case reports. The genomic profiles of patients with cancer are typically presented in genetic testing reports, which contain information on potential actionable targets with variant classification based on well-recognized guidelines and recommendations. However, clinicians were generally unfamiliar with the variant classification criteria, such as the 4-class systems for somatic mutations [49] and the five-class system for germline variants [44], and their clinical significance. Therefore, using a straightforward and clinically oriented way to display genetic results may help improve the effectiveness of the decision-making process. Furthermore, each clinical evidence for a given genomic variant is often presented separately in genetic testing reports, whereas clinicians often prefer a more integrated and comprehensive summary that synthesizes multiple related pieces of evidence, even when discrepancies exist.

In addition, most clinicians express a need for support with feasibility considerations in the diagnosis and treatment process. Although current drug insurance and CT information query systems are well developed, their integration with patient information is insufficient. Future feasibility-support PODS tools should integrate patient information from local or in-house hospital information system data with medication use, making it easier for clinicians to access relevant data. In addition, diagnostic and prognostic algorithms or models have broad potential applications in clinical practice, supporting clinicians in optimizing the decision-making process in various situations [45,50].

Support in the decision-making process is a less developed yet widely demanded aspect of existing PODS tools. Clinicians are interested in improvements in integrating patient information, optimizing information visualization, and enhancing search and recommendation methods [51] for a more efficient decision-making process. In particular, oncology knowledge–related PODS tools should feature an optimized recommendation function [52] and a matching algorithm based on the level of evidence and CTs to enhance clinicians’ confidence in using these tools [53]. With the development of language models, knowledge acquisition through question answering or dialogue is also expected to become a future direction for knowledge-related PODS tools [54]. Currently, the integration of patient information in PODS tools is limited and often requires manual input [55]. Automatic integration of patient information and improved visualization would offer significant assistance in MDT meetings and routine clinical practice [10,56].

When adapting the framework to hospitals of different sizes, resource levels, and IT infrastructures, some previous experiences and frameworks may bring value to the development and deployment of a system in a real environment. The Nonadoption, Abandonment, Scale-Up, Spread, and Sustainability (NASSS) framework for complex health technologies will assist in predicting and evaluating the success of a PODS deployment [57]. The Exploration, Preparation, Implementation, and Sustainment framework will be a guideline in PODS implementation [58].

### Comparison With Prior Work

PODS can be understood as a specialized form of a clinical decision support system (CDSS). Our work directly builds upon a substantial body of former research. The model by Hendriks et al [59] identified critical barriers, including guideline maintenance and electronic health record interoperability, aligning with our findings on the necessity of dynamic knowledge updating. However, our work extends this paradigm by systematically addressing 3 dimensions in PODS.

Walsh et al [17] examined existing oncology-focused CDSS platforms, identifying critical dimensions, such as data integration, AI capabilities, and stakeholder concerns, and highlighting how technical sophistication alone is insufficient without addressing clinicians’ real-world concerns about data quality, user burden, and trust in AI-driven recommendations. Liao et al [60] documented the rapidly growing intersection of AI with clinical oncology workflows, suggesting that advanced natural language interfaces could improve information retrieval and decision support in tumor boards.

Since Kurnit et al [7] proposed the vision of PODS for harnessing genomic and molecular testing, alongside targeted therapy and immunotherapy insights to enable precision oncology at the point of care in 2018, a variety of PODS tools have emerged, driven by advances in next-generation sequencing, biomarker-driven therapeutics, and data-sharing initiatives [23,24,28,61,62]. However, despite these technological strides, there is no comprehensive synthesis of what characteristics and functions PODS should offer or a clear mapping of clinicians’ practical needs for such systems.

In contrast to prior studies that have predominantly focused on theoretical frameworks or technical prototypes, our study adopts a descriptive and user-centered approach. Through intensive qualitative observations of MDT discussions and in-depth interviews with clinicians of varied specialties and experience levels, we systematically identified and categorized the functional requirements of PODS.

### Strengths and Limitations

Our contribution complements existing CDSS and PODS literature by moving beyond prototype descriptions and generic recommendations, instead offering a pragmatic, empirically derived framework for future PODS development that aligns tightly with the lived experiences of oncology practitioners.

However, the needs analysis should be considered in light of certain study limitations. First, the rapid development of precision oncology means that additional needs may arise in the future. Convening a multidisciplinary advisory group from different specialties from time to time to evaluate emerging technologies, therapies, and policy changes as stakeholder review cycles occur and using structured Delphi rounds to reach consensus on necessary revisions may help maintain a living functional needs framework for PODS, which could be applied to integrate perspectives from different departments to validate the prioritization outcomes. Moreover, the MoSCoW (must have, should have, could have, won’t have) method is effective in distinguishing critical needs from optional ones during the PODS implementation phase. Second, this study was conducted in only 1 hospital in China, highlighting the need for broader investigations across more regions and clinical units. In addition, a follow-up survey across a larger sample and additional quantitative validations may help validate the identified needs and their relative importance. In practice, institutions can conduct a rapid workshop with local stakeholders to map existing workflows, data systems, and patient populations against the 3 framework dimensions proposed before PODS implementation. Prioritizing the needs when implementing PODS tools is also needed for further study. Third, the observation and interview processes were based on the subjective perspectives of researchers and interviewees, which may introduce some bias. While the study focuses on clinicians, the needs framework may change when considering other stakeholders (eg, hospital administrators and IT developers). A bigger study integrating their perspectives will be needed in the future. Future quantitative studies—such as developing questionnaires focused on areas of likely interest to nonclinical stakeholders, including cost management, data interoperability, IT infrastructure readiness, and organizational feasibility—can help identify key domains and contribute to broader integration, potentially leading to new thematic categories within the framework. Fourth, clinicians may express various needs for specific content, such as CTs, which might be dispersed across these 3 themes. We acknowledge that this is unavoidable. Nonetheless, we anticipate that this classification will provide a new perspective for PODS system developers, offering a way to consolidate the diverse needs of clinicians.

### Future Directions

Although the development of PODS tools is rapid, the traditional genetic testing report will likely remain an irreplaceable support for genomic interpretation well into the future. The combination of the genetic testing reports and PODS tools may offer complementary advantages, providing new paradigms in oncology decision-making, which could be explored in further research. Advancements in AI, such as generalist models or large language model agents, may provide improved optimization and interaction capabilities for clinicians and PODS tool developers.

### Conclusions

The rapid advancement of precision oncology has created an increasing demand for decision support tools to assist clinicians in navigating complex treatment choices. This study provides valuable insights into the current landscape of PODS tools, identifying and categorizing the needs and challenges that clinicians face in using these tools effectively. Our findings highlight key areas for improvement, including better access to oncological knowledge, clinical contextualization and resource navigation, as well as support abilities in the decision-making process. In addition, we propose a functional framework that outlines the essential features of an integrated PODS tool, which could enhance clinical decision-making in precision oncology. The results offer a foundation for future research and development, emphasizing the need for tools that are not only technologically advanced but also aligned with the practical needs of clinicians in diverse health care settings.

## Appendix

Multimedia Appendix 1 Supplementary materials.

## Abbreviations

AI: artificial intelligence
CDSS: clinical decision support system
COREQ: Consolidated Criteria for Reporting Qualitative Research
CT: clinical trial
MDC: Molecular Diagnostic Center
MDT: multidisciplinary team
MoSCoW: must have, should have, could have, won’t have
NASSS: Nonadoption, Abandonment, Scale-Up, Spread, and Sustainability
PODS: precision oncology decision support
PUCH: Peking University Cancer Hospital

## Data Availability

The datasets generated or analyzed during this study are not publicly available due the privacy of patients and physicians in the structural observation and in-depth interviews, but are available from the corresponding author on reasonable request.
