# Supplementary material for: Need Analysis of Clinician-Oriented Integrated Precision Oncology Decision Support Tools: Qualitative Descriptive Study
Source: JMIR Hum Factors. 2025 Sep 8;12:e67476. doi: 10.2196/67476 (PMC12455164; doi:10.2196/67476)
Supplement: Multimedia Appendix 1 [file humanfactors_v12i1e67476_app1.docx]

**Supplementary Material 1 Multidisciplinary Team Discussion QO Record**

Note: In the first 2 weeks of MDTs, ZS & FY only recorded the summaries of the discussions of different clinicians and patients’ basic information. As he became more familiar with the identities of the participants (about 3-4 weeks), ZS felt that it was necessary to record the duration of the discussion and further standardize the record content. After discussion with FY, this QO Record format was made, and all situations that meet the needs of the study could be recorded.

| **Temporal Information** | | |
| --- | --- | --- |
| Date: | Start Time: | End Time: |
| **Patient Information** | | |
| Age: | Sex: | |
| Cancer Type: | TNM Staging: | Staging: |
| Symptoms: | | |
| Genetic Testing Result: | | |
| **The Purpose of Discussion:** | | |
| **The Presentation of Clinicians** | | |
| Surgeons & Radiotherapists | Number: | **Comments and Possible Requirements in PODS Systems** |
| Content (Record per person, can be added repeatedly similarly hereinafter) | |  |
| Physicians | Number: |  |
| Content | |  |
| Radiologists | Number: |  |
| Content | |  |
| MDs & Pathologists | Number: |  |
| Content | |  |
| Nursing, TCM & Other Clinicians | Number: |  |
| Content | |  |
| **The Results of Discussion & Treatment Decision:** | | |

**Supplementary Material 2 The Process of MDT and Pattern of Using Genetic Test Reports in Peking University Cancer Hospital (PUCH)**

**1. The qualitative observation of Current status of PODS tools’ usage and decision making in MDT**

After six months of observation, we found that the discussion and decision-making processes varied based on the purpose of the discussion, which depended on the patient's condition (Table S1).

In *Preparation*, chief residents in Ward from Thoracic Oncology Center are normally the one that take in charge of medical record preparation after a list of referral patients for MDT discussion raised by clinicians. Medical history, courses of disease and treatments were collected from different database, such as HIS, LIMS, PACS, followed by summarization in slides for briefing in MDT discussion. Patient ID and brief summary of medical records were sent to MDT participants in advance, in order to help them preview patient examination results and refer to relevant knowledgebase and database accordingly. Molecular biologists and geneticists may collect supportive evidences from knowledgebases searching and literature review for genomic variant classification and the interpretation of clinical significance based on patient genetic testing results. The less common of genomic variants, the more challenge and time consuming of evidence collection. Therefore, the preparation time of genetic testing results interpretation may vary a lot, from 15 minutes to 1 hour, due to the number of variants to be check, evidence searching methodology, the richness, depth, and variety of knowledge-base, etc. Clinician may refer to available CT when standard treatments failed and/or neither guideline nor convinced clinical evidence could be followed. However, CT searching process are also time consuming and labor intensive. PUCH in-house CT will be filtered firstly, followed by searching available CT in ClinicalTrials.gov and ChiCTR.

During *Introduction*, patient slides and prepared treatment options were presented. After medical case briefing, radiologists firstly assess and interpret medical imaging examination results and provide professional suggestions on staging and therapeutic efficacy evaluation. Surgeons, physicians, and radiotherapists then give recommendations on treatment plans based on clinical diagnosis, clinical benefit, and potential prognosis, whereas pathologists, molecular biologists and geneticists normally give diagnostic and treatment proposals according to histopathological results, molecular pathological results and genetic testing results. The characteristics of the records in the discussion of MDT in Thoracic Oncology Center are described in Table S1. Disagreements were resolved by voting, and the results were shared with the patient to finalize the treatment decision.

Table S1 Characteristics of the records in the discussion of MDT in Thoracic Oncology Center.

| **Number of Records** | 86 |
| --- | --- |
| **Number of Patients with Genetic Testing (%)** | 39 (45%) |
| **Number of Patients with Clinical Significant Molecular Alteration** | 24 (28%) |
| **Topic of Discussion (%)** |  |
| Establishing Diagnosis | 20 (23%) |
| Select Treatment Options | 73 (85%) |
| **Stage (%)** |  |
| Pathology Unknown | 16 (19%) |
| Early Stage | 25 (29%) |
| Advanced Stage, Stable | 24 (28%) |
| Advanced Stage, In Progress | 21 (24%) |
| **Other Significant Features (%)** |  |
| Multiple Lesions | 6 (7%) |
| Advanced Age (>80) | 6 (7%) |
| Severe Complication | 10 (12%) |
| Involved in Other Cancer Type | 7 (8%) |
| **Guideline Assistance (%)** |  |
| Available | 54 (63%) |
| Executed | 23 (27%) |
| Unspecified | 9 (10%) |
| **Discussion Result (%)** |  |
| Further Diagnosis & Test | 14 (16%) |
| Surgery | 11 (13%) |
| Radiotherapy | 9 (10%) |
| Chemotherapy | 12 (14%) |
| Chemoradiotherapy | 6 (7%) |
| Targeted Therapy | 15 (17%) |
| Immunotherapy | 15 (17%) |
| Participate in Clinical Trials | 7 (8%) |
| TCM & Palliative Care | 2 (2%) |
| Surveillance & Follow-up | 8 (9%) |
| Manage Complication Only | 5 (6%) |

**2 The behavioral pattern of using genetic test reports**

There were differences between physicians and surgeons in how they used genetic test reports. Surgeons, who focused on surgical aspects, were less knowledgeable about targeted drugs than physicians. Therefore, they paid more attention to the summaries of guideline-recommended targeted drugs in the genetic testing reports for perioperative adjuvant therapy. Physicians, experienced on drug use and familiar with systematic therapy guidelines, read the reports faster and paid more attention on summaries of clinical significances and potential therapies of the variation described in guidelines, conferences and consensuses. Chemotherapy drug evidence related to genetic tests was seldom emphasized, as it largely did not affect their chemotherapy decisions.

*“… we usually don’t consider other chemotherapy related genes than UGT1A1 … even if there is side effect, we can either alleviate them or change to another chemotherapy option …”* *(008, associate chief physician)*

**Supplementary Material 3 Semi-structured Interview**

**The Usage and Needs in Precision Oncology Decision Support Tools**

**Semi-structured Interview Guide**

**Demographic Questions**

1. What is your role in the routine clinical practice of oncology?
2. How many years of clinical experience do you have?
3. What is your role in multidisciplinary team?
4. Do you have experience using genetic test report?
5. Do you have experience using precision oncology decision support tools, like knowledge bases, clinical trial databases, therapy recommendation engines, literature search tools or predictive algorithm-based models etc.?

**Interview questions in the usage of PODS tools**

1. How well do you know precision oncology decision support tools?
2. How do you use these tools at work, especially in routine clinical practice and multidisciplinary team?

Probe: What PODS tools do you use? Do you use any interactive PODS tools before MDT?

1. How well do these tools support you in cancer diagnosis and treatment?
2. How do you hope these tools will help you in your cancer diagnosis and treatment decisions?

Probe: What kind or kinds of PODS tool interest you most? Why?

1. How do you hope these tools will help the multidisciplinary team discussion?

Probe: What function, change or alteration in PODS tools do you think will help improve the quality of the discussion? Why?

**Interview questions in the functional needs of PODS tools**

1. How well do you need oncological knowledge support in decision making? What kind of knowledge do you think you may need?
2. Besides the oncological knowledge, are there any other functions or needs do you think in assisting you implementing the diagnosis or treatment functions?
3. In MDTs and clinical routine practice, what other functions or needs do you think that PODS tools should reach or satisfy?

**Supplementary Material 4 Supplementary Tables**

Table S2 Themes emerged from the observation records in the discussion of MDTs.

| **theme** | **Number of MDTs** | **Reference point** | **remark** |
| --- | --- | --- | --- |
|  |  |  |  |
| **TAG-Oncological Knowledge-Treatment** |  |  |  |
| **Discussion in update & changes in guidelines, conferences and consensuses of thoracic cancer** |  |  |  |
| Discussion of guidelines | 25 | 32 |  |
| Discussion of conferences | 14 | 16 |  |
| Discussion of consensuses | 4 | 4 |  |
| **Share of CT information** |  |  |  |
| Confirmation if there are new CT available | 35 | 42 |  |
| Discussion of halfway result of a particular CT | 8 | 14 |  |
| **Discussion about therapy options & drugs** |  |  |  |
| Usage & dosage | 11 | 12 |  |
| Side effects | 4 | 4 |  |
| Comparison of effects among the options | 62 | 87 |  |
| **Discussion of complex cases** |  |  |  |
| Needs of multidisciplinary discussion from other oncological departments | 7 | 10 |  |
| Consult of similar experience or case reports | 15 | 15 |  |
| **TAG-Oncological Knowledge-Diagnosis & Prognosis** |  |  |  |
| **Function of biomarkers & molecular alteration** | 8 | 8 |  |
| **Oncogenecity & pathogenecity** | 15 | 15 |  |
| **Prognosis** | 13 | 16 |  |
| **TAG-Clinical Contextualization and Resource Navigation** |  |  |  |
| **Accessibility of CT** |  |  |  |
| Confirmation if there are new CT available (see Share of CT information) | 35 | 42 |  |
| Confirmation of the recruitment status | 6 | 6 |  |
| **Accessibility of drugs** |  |  |  |
| Discussion of the patient's affordability | 16 | 16 |  |
| Disccusion the accessibility or cover by Chiniese medical insurance policy | 9 | 9 |  |
| **Necessity of ordering diagnostic tests** |  |  |  |
| Puncture | 21 | 21 |  |
| Surgical biopsy | 12 | 12 |  |
| **TAG-Other Support Functions may be Implemented by PODS Tools** |  |  | This part was concluded through the observers' intuitive feeling, not analysed in the discussion of clinicians. |
| **Integration of Patient Information** |  |  |  |
| HIS |  |  |  |
| LIS |  |  |  |
| PACS |  |  |  |
| LIMS |  |  |  |
| **Better Visualization** |  |  |  |

Table S3 Themes emerged from the in-depth interviews. The themes in italics means they emerged only in interviews.

| **theme** | **Number of interviewees** | **rp of all** | **rp of physicians** | **rp of surgeons & radiotherapists** | **rp of MDC** |
| --- | --- | --- | --- | --- | --- |
|  |  |  |  |  |  |
| **TAG-Oncological Knowledge-Treatment** |  |  |  |  |  |
| **Timely notification of update & changes in guidelines, conferences and consensuses of thoracic cancer** | 12 |  |  |  |  |
| Guidelines | 11 | 15 | 8 | 5 | 2 |
| Conferences | 8 | 10 | 7 | 2 | 1 |
| Consensuses | 1 | 1 | 1 |  |  |
| **Automatic updating CT information** | 14 |  |  |  |  |
| Needs of enrollment & treatment options in CTs | 14 | 14 | 9 | 4 | 1 |
| Halfway results | 6 | 6 | 6 |  |  |
| **Information of therapy options & drugs** | 8 |  |  |  |  |
| Usage & dosage | 7 | 7 | 5 | 2 |  |
| Side effects | 4 | 4 | 4 |  |  |
| **Knowledges for complex cases** | 4 |  |  |  |  |
| Multidisciplinary guidelines | 1 | 1 | 1 |  |  |
| Case report | 4 | 4 | 4 |  |  |
| **TAG-Oncological Knowledge-Diagnosis & Prognosis** | 6 |  |  |  |  |
| **Function of biomarkers & molecular alterations** | 4 | 4 | 1 | 2 | 1 |
| **Oncogenicity & pathogenicity** | 5 | 5 | 4 |  | 1 |
| **Prognosis** | 4 | 4 | 1 | 3 |  |
| **TAG-Clinical Contextualization and Resource Navigation** |  |  |  |  |  |
| **Accessibility of CT** | 9 |  |  |  |  |
| Inclusion/exclusion criteria | 6 | 9 | 6 | 3 |  |
| *Geographic information of CT* | 6 | 8 | 8 |  |  |
| Recruitment status | 8 | 8 | 5 | 3 |  |
| **Accessibility of drugs** | 5 |  |  |  |  |
| Accessibility or cover by Chiniese medical insurance policy | 5 | 5 | 5 |  |  |
| **Necessity of ordering diagnostic tests & prediction of prognosis** | 4 |  |  |  |  |
| Puncture & surgical biopsy | 3 | 3 |  | 2 | 1 |
| Prognosis support from prediction model | 2 | 2 |  | 2 |  |
| **TAG-Support Abilities Demands in Decision-Making Process** |  |  |  |  |  |
| **Integration of Information** | 8 |  |  |  |  |
| Integration of patient information | 5 | 8 | 4 | 3 | 1 |
| *Recordability & traceability of MDT records* | 6 | 9 | 8 | 1 |  |
| **Better Visualization** | 3 | 3 | 1 | 1 | 1 |
| ***Using richer biological knowledge and phenotypes as search criteria*** | 10 |  |  |  |  |
| *Incorporate biological knowledge in information searching* | 4 | 5 | 5 |  |  |
| *Combine phenotypes in information searching* | 9 | 11 | 6 | 4 | 1 |
| ***Optimization in retrieval, recommendation & question answering*** | 5 |  |  |  |  |
| *Treatment options recommendation* | 5 | 6 | 5 |  | 1 |
| *Question answering, artificial intelligence & LLM* | 2 | 4 | 2 | 1 | 1 |

Table S4 The percentage of needs of PODS tools from in-depth interview.

| **Needs** | **total** | **percentage** |
| --- | --- | --- |
| Better Visualization of information | 3 | 17.65% |
| Multidisciplinary guidelines and similar case report for complex cases | 4 | 23.53% |
| The necessity of ordering diagnostic tests and prediction of prognosis | 4 | 23.53% |
| The accessibility of drugs | 5 | 29.41% |
| Optimization in retrieval, recommendation and question answering | 5 | 29.41% |
| Knowledge support in diagnosis and prognosis | 6 | 35.29% |
| The usage of drugs | 8 | 47.06% |
| Automatic integration of patient information | 8 | 47.06% |
| The accessibility of CTs | 9 | 52.94% |
| Using richer biological knowledge and phenotypes as search criteria | 10 | 58.82% |
| Timely notification for updates and changes in conferences, guidelines and consensuses | 12 | 70.59% |
| Automatic updating CT information | 14 | 82.35% |
| Knowledge support in therapies | 17 | 100% |

Table S5 The characteristics of the interviewee. Preparer, the chief residents taking in charge of medical record preparation.

| **No.** | **Professional Title** | **Job Role** | **Sex** | **Work year** | **Preparer** | **Had used at least one of PODS tools** |
| --- | --- | --- | --- | --- | --- | --- |
| 001 | Attending Chief | Surgeon | M | 5 | √ |  |
| 002 | Attending | Surgeon | M | 2 | √ | √ |
| 003 | Chief | Surgeon | M | 24 |  | √ |
| 004 | Attending | Physician | F | 3 | √ | √ |
| 005 | Associate Chief | Physician | F | 17 |  |  |
| 006 | Associate Chief | Physician | F | 16 | √ | √ |
| 007 | Chief | Physician | M | 25 |  | √ |
| 008 | Associate Chief | Physician | F | 16 |  | √ |
| 009 | Associate Chief | Surgeon | M | 22 |  |  |
| 010 | Attending | Physician | M | 4 | √ | √ |
| 011 | Attending | Physician | F | 3 | √ | √ |
| 012 | Associate Chief | Physician | F | 14 |  |  |
| 013 | Associate Chief | Surgeon | M | 15 |  |  |
| 014 | Supervising | Molecular Biologist | M | 11 |  | √ |
| 015 | Associate Chief | Physician | F | 18 |  |  |
| 016 | Deputy Chief | Molecular Biologist | M | 15 |  | √ |
| 017 | Attending Chief | Radiotherapist | M | 6 |  |  |

**Table S6** Sources of themes from key informants, translated into English.^a^

| Theme and subtheme | | | Illustrative quote and quote number |
| --- | --- | --- | --- |
| **Better access to oncological knowledge** | | | |
|  | **Knowledge support in therapies** | | |
|  |  | Guidelines, conferences, and consensuses | - “Before the MDT, Dr. [name] would like to share *recent progress* of [progress in treatment of specific disease]...” [Observation 008, 028, and 079] (Quote 1) - “The way I get access to the oncological knowledge are usually from WeChat official accounts, going to *conferences*, and *newly updated information* shared by peer expertise. Sometimes I also search for *literatures or reviews* in my spare time...What I’m most interested in is the change of the *latest version update guideline*, from which I could learn a lot...would be great if there is a *notification of the changes*...” [005, associate chief physician] (Quote 2) - “We usually discuss the *latest progress of conferences* before the MDT starts. If PODS tools could *notify us this information* in our spare time, it would help a lot in *summarizing this information* for discussion...” [006, associate chief physician] (Quote 3) |
|  |  | Clinical trials | - “About 25-50% of patients in our hospital would require *enrollment in CTs...*develop *a* *real-time updating CT recommendation tool* is the best...” [004, attending physician] (Quote 4) - “...The information in the website of CTs updates frequently...requires us to *retrieve and summarize the latest eligibility criteria and enrollment* every half month to one month...so time-consuming...” [009, associate chief surgeon] (Quote 5) - “...*Latest progresses* are often discussed in MDT...not so convenient to check...we want future PODS tools could capture these in real time...” [005, associate chief physician] (Quote 6) - “Before the MDT, Dr. [name] would like to share the *midterm result* of [progress of specific CT] for us...” [Observation 033 and 082] (Quote 7) |
|  |  | Drugs and treatment options | - “Genetic test reports that vormetinib is effective in LUAD patients with EGFR exon 20 insertion mutation...*the descriptions are not particularly clear*...we sometimes want to *understand the mechanism of drugs*...” [003, chief surgeon] (Quote 8) - “Osimertinib, vormetinib and almonertinib are commonly used among the third generation of EGFR-TKIs...there are *differences in their use*, which requires years of clinical experience...We hope PODS tool will support us in this regard...” [010, attending physician] (Quote 9) - “...Some drugs are needed for four cycles, but some patients’ health condition is poor...We want to know *whether can I reduce the dosage*...sometimes drug suppliers would tell us some information, but that’s not enough...” [005, associate chief physician] (Quote 10) - “...The two most common and risky *side effects* are vomiting and leukopenia...need prompt intervention...if PODS tools could have a *alert or reminder function of these side effects*, it would help us reduce unnecessary prescriptions to alleviate these side effects...” [001, associate chief physician] (Quote 11) |
|  |  | Knowledge for complex cases | - “...when the patient has other cancers...we would invite colleagues from the corresponding departments for MDT discussion, but it is brief...If we can learn easily from *other disciplines*, we can make treatment decisions more quickly...” [006, associate chief physician] (Quote 12) - “Today’s discussion is about the patient with malignant pleural mesothelioma for whom conventional chemotherapy drugs did not respond...if any doctors have seen *relevant case reports*?” (Observation 046) (Quote 13) |
|  | Knowledge support in diagnosis and prognosis | | - “...some colleagues asked me the *differences between germline variants and somatic mutations*...PODS tools could be a useful way to help my colleagues understand *the biological knowledge* like this...” [014, supervising molecular biologist] (Quote 14) - “...we sometimes want to know *the biological function of molecular alterations*, understanding why the alteration happens could better guide us to understand the mechanism of the disease...” [013, associate chief surgeon] (Quote 15) - “...previously encountered a LUAD patient with co-mutations in TP53, RB1, and EGFR...*no appropriate standard treatment or diagnosis options* but continued observation at the time...*an article* said that the concurrence of three variants may be induced to small cell cancer transformation, which is an important reference basis to do puncture for diagnosis...hope to have a better mechanism to search evidences...” [011, attending physician] (Quote 16^b^) - “...we often *evaluate the prognosis between different therapies*, like radiotherapies and targeted therapies...the knowledge of which could help us make decision better...” [017, attending chief radiotherapist] (Quote 17) |
| **Clinical contextualization and resource navigation** | | | |
|  | Accessibility of CTs^c^ | | - “When there is a patient needs to be enrolled, we will look at *the inclusion and exclusion criteria in the CTs* we have printed (take out some hard copy of CTs) and compare the patient’s medical status with the criteria item by item...it is very time consuming...Our *CT screening* usually follow this sequence: ‘First, CTs held in our ward; Second, CTs held in our hospital; Third, CTs held in Beijing’s Hospital; Fourth, different places other than Beijing in China; Lastly, we consider overseas’...CT enrollment is usually based on the *screening order* of inside the department, hospital, Beijing, China and foreign countries...We need to manually screen it ourselves...want *an automatically sort*...” [002, attending surgeon] (Quote 18) - “Sometimes we know there is an enrollment of CT, but we don’t know the *up-to-date recruitment status*. Future PODS tools or CT website should *update the status more frequently*.” [006, associate chief physician] (Quote 19) |
|  | Accessibility of drugs | | - “...Osimertinib is covered in *Drug Catalog*, but vormetinib and almonertinib need to be paid at patients’ own expense...” [001, associate chief physician] (Quote 20) - “*Clear mark of drug catalog and price information* of drugs can make us discuss the most suitable treatment option for with patient him/herself.” [012, associate chief physician] (Quote 21) |
|  | Prediction models: the necessity of ordering diagnostic tests and prediction of prognosis | | - “...some genetic tests are expensive...I have known that there are some radiology technologies to auxiliary identify whether there is a certain *risk of mutations*...” [012, associate chief physician] (Quote 22) - “...examinations like puncture examinations are invasive...we want to use PODS tools to help *estimate the necessity of such invasive examinations*...” [013, associate chief surgeon] (Quote 23) - “...common mutations like TP53 have a poor prognosis...We want to *combine the molecular alteration with prognostic prediction*...” [002, attending surgeon] (Quote 24) |
| **Support abilities in the decision-making process** | | | |
|  | Using more flexible biological knowledge and phenotypes as search criteria | | - “...we once had a patient with MEK mutation and ERK wild type...there were not many drugs targeting MEK...wanted to find some CTs targeting ERK...had to reset the search criteria which was inconvenient...would like to have *a flexible retrieval mechanism*...” [008, associate chief physician] (Quote 25) - “...would be great if PODS tools could *automatically match the inclusion criteria with patient’s information* and tell us directly whether this patient is a qualified candidate...helps us reduce a lot of comparison work...” [007, chief physician] (Quote 26) |
|  | Automatic integration of patient information | | - “...would be great if we *just input* the ID of patient and *click a button*...all the information scattered in HIS, PACS...is *organized*...” [011, attending physician] (Quote 27) - “Acquiring the radiology information is inconvenient currently. We have patients all over the country, and they have a variety of formats and types of image information. We hope that the future of PODS tools could help us to *reduce the burden of identification and input of these data*.” [010, attending physician] (Quote 28) - “Suppose there is a *timeline of patient* in MDT, and all the events of this patient are on the line. Click one button and the details of the event will be shown. That’s amazing...” [002, attending physician] (Quote 29) - “Some patients will be discussed for several times. We want the PODS tools to *record our discussion every time*.” [004, attending physician] (Quote 30) - “This patient was discussed [time] ago. Last time we decided to use [treatment option], but the disease progressed now...” [Observation 033] (Quote 31) |
|  | Better visualization of information | | - “...Switching slides back and forth to watch the texts is inconvenient...hard to *sort out the therapeutic relationships*...” [010, attending physician] (Quote 32) - “...*automatically generated timeline of history* is more logical and visualizable...” [002, attending surgeon] (Quote 33) - “...when there is a patient with rare mutation...may want to *find previous patients who have the same or similar mutation*...may be helpful for diagnosis and treatment...some simple *automated generated statistical distribution plots* are intuitive to see and discuss...” [014, supervising molecular biologist] (Quote 34) |
|  | Optimization in retrieval, recommendation, and question answering | | - “...would be great if PODS tools could *automatically match the inclusion criteria* with the patient information...” [005, associate chief physician] (Quote 35) - “...don’t have enough time to read and search the evidences...we may just input some key search information to *AI*, and ask what latest information it finds...” [015, associate chief physician] (Quote 36) - “I wonder if there’s a day if *AI could be a real assistant* for me. Nowadays there is chatGPT, I believe in future there will be *a chatbot in oncology area*, knows everything, tells us what is the best treatment option...future PODS tools may be like this...” [002, attending surgeon] (Quote 37) |

^a^The descriptive codes for themes, subthemes, and subcategories in the illustrative quotes were overstruck.

^b^The interviewee referenced the study by Lai et al [45].

^c^CT: clinical trial.

The percentages in brackets in the sections below represent the percentage of interviewees who reported the corresponding functional requirements. The percentages reflect only the proportion of participants who mentioned a specific need during the interviews and do not indicate the relative importance of the needs.

Table S7 The functional framework of needs for integrated PODS^a^ tools.

| Dimension and subcomponents | | Functions of needs | Recommendation |
| --- | --- | --- | --- |
| **Up-to-date and straightforward oncology knowledge** | | | |
|  | High-level evidence | - Access to high-level clinical evidence (eg, guidelines, conferences, and consensuses) - Concise overview of evidence, containing a general idea of medical information (eg, drug, therapy, and test results) - Labeling of credibility or describing the clinical significance or referability - Source of literature | High-level evidence (eg, guidelines, conferences, and consensuses) is always a priority for clinicians when making decisions about any patient. Clear labeling of the evidence level is also helpful for clinicians in determining the credibility of the evidence [46]. |
|  | CTs^b^ | - Information on inclusion and exclusion criteria (related to the accessibility of the CT function) - Provide midterm results, if available, for CTs that are ongoing and not recruiting - Provide enrollment information for recruiting CTs - Comprehensive description of medication information as outlined in the CT protocol | After the failure of guideline-recommended therapies, appropriate CTs should be considered. |
|  | Biological knowledge in diagnosis, treatment, and prognosis | - Description of basic biological information of biomarkers, such as nomenclature (eg, alias and transcript numbering), type (eg, point mutation, fusion, and copy number variation), structural information (eg, mutation site and location in chromosome), and basic biological function - Description of the medical function of biomarkers related to oncology, such as the role in tumor development (eg, oncogenic or tumor suppressor and driver or passenger), mechanism of involvement in tumor development (eg, kinase activation and DNA damage repair), and frequency of expression and mutation in different cancers - Description of the medical function of biomarkers directly related to patient information, such as clinical significance (eg, diagnosis, treatment, and prognosis) and pathogenicity - Description of inclusion in public evidence repositories of biomarkers - Source of evidence | A better explanation of biological knowledge will help clinicians understand the pathological mechanism. The integration will also improve the interpretation of the clinical significance of genomic testing results [47]. |
|  | Drug and therapy information | - Information on the use, dosage, and side effects of drugs and therapy options from prior medication and literature | The use, dosage, and side effects information of drugs and therapy options help clinicians implement treatment accurately. |
|  | Case reports and preclinical evidence | - Source of the case reports related to the patient - Phase 1 to 2 CTs and animal experiments of drugs | Case reports and preclinical evidence are important references for complex or rare cases. |
|  | Multidisciplinary guidelines | - Clinical guidelines for different cancer areas | Although the proportion of patients with multiple primary cancers is small, the number of discussions about these patients is gradually increasing. These guidelines can be used to keep physicians updated on other cancer types outside of MDTs. |
| **Clinical contextualization and resource feasibility support** | | | |
|  | Accessibility of CTs | - Automatically match patient information with inclusion and exclusion criteria (refer to the information on the inclusion and exclusion criteria function) - Automatically estimate recruitment status - Provide an automatic geographic accessibility for eligible CTs | For patients, the enrollment criteria of CT are as important as the therapy itself. Letting the clinician know whether a certain CT could be accessed also matters. |
|  | Accessibility of drugs and treatment options | - Information on the insurance coverage status of drugs - Cost of drugs and treatment options - Approval information of drugs and treatment options | This function helps clinicians know about drug accessibility and could save time in screening available CTs and communicating with patients. |
|  | Application of prediction models | - Convenient interface to access prediction models | There are many prediction models in the oncology domain, such as operative risk assessment and risk of recurrence. However, the current PODS tools have not integrated these models yet. Integrating and applying these models could be helpful in decision-making. |
| **Comprehensive and efficient support abilities in the decision-making process** | | | |
|  | Individualized search criteria | - Incorporate biological knowledge (eg, the function of variants or signaling pathways) - Incorporate phenotypic features (eg, stage, number of treatment lines [ie, the treatment course], and the location of the lesion) into search criteria | Optimization in search criteria, such as biological knowledge and phenotypes, could help clinicians search for treatment options more precisely. |
|  | Automatic integration of patient information | - Automatically summarize the patient’s diagnosis and treatment history - Automatically integrate the patient’s health information from different systems in the hospital, such as the HIS^c^, PACS^d^, and LIS^e^. | The automatic summarization of patient information could help reduce the *preparation* time. |
|  | Recordability and traceability | - An interface for recording MDT^f^ discussion, preferably automatically | A timely recording of the MDT discussion function could help clinicians trace back, summarize, and analyze previous data easily. |
|  | Better visualization | - Chronological patient history - Logical display - Organization of information by priority - Clear hierarchy of information | Automatically generated timelines help clinicians read patients with a long history and complex treatment course straightforwardly, whereas graphs and tables can help them understand data better and interpret clinical significance more easily. |
|  | Treatment option recommendation | - Personalized ranking of drugs and treatments based on their advantages and disadvantages - Provide automated treatment recommendations | A treatment recommendation function with evidence classification, data matching, and a sorting algorithm could provide extra comprehensive support to clinicians during decision-making. |
|  | Question answering and dialogue | - Automatic dialogue and question answering - Large language model agents that automatically retrieve information or tools to support decision-making | The development of artificial intelligence and large language models may implement the function of multiturn question answering and dialogue, as well as provide precise information. |

^a^PODS: precision oncology decision support.

^b^CT: clinical trial.

^c^HIS: hospital information system.

^d^PACS: picture archiving and communication system.

^e^LIS: laboratory information system.

^f^MDT: multidisciplinary team.

**Supplementary Material 5 Supplementary Figures**

**
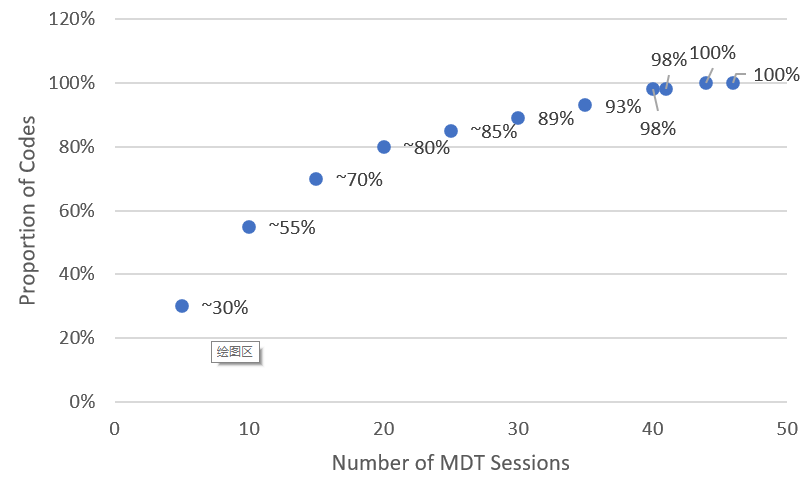
**

Figure S1 Proportion of codes in 5,10,15,20,25,30,35,40,41,44,46 MDT sessions. Data saturation was reached in the 41^st^ MDT, and no more new codes emerged in the 45^th^-46^th^ MDT.

**
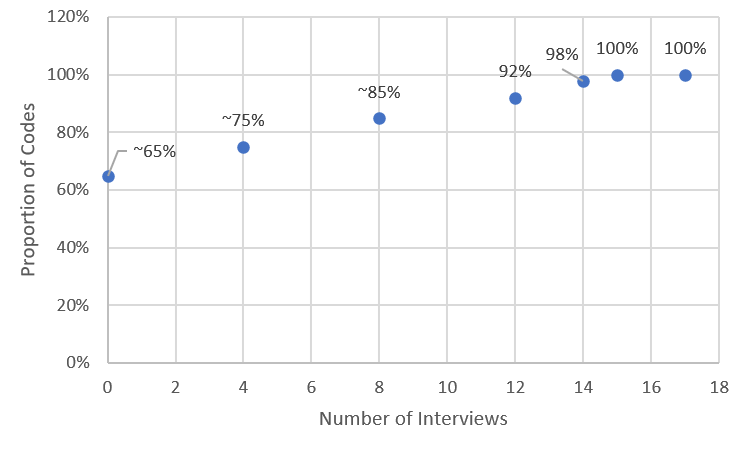
**

Figure S2 Proportion of codes in 0,4,8,12,14,15,17 interviews. At the base of MDT observation, 65% of codes was recorded before the interviews. Data saturation was reached in the 14^th^ interview, and no more new codes emerged in the 15^th^-17^th^ interview.

**
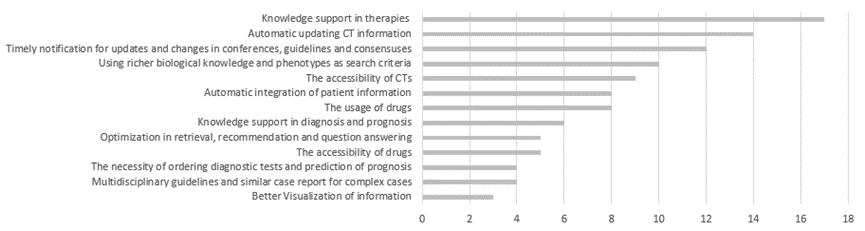
**

**
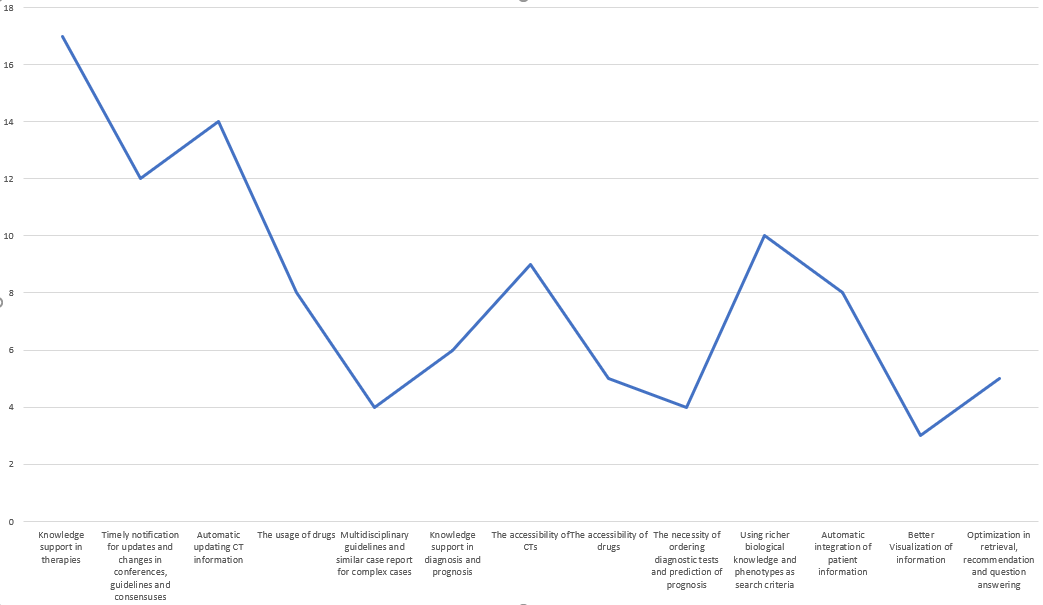
**

Figure S3-S4 The themes, subthemes and the number of interviewees who mentioned the it. CT, clinical trial.
